# Multimodality treatment of brain metastases: an institutional survival analysis of 275 patients

**DOI:** 10.1186/1477-7819-9-69

**Published:** 2011-07-05

**Authors:** Ameer L Elaimy, Alexander R Mackay, Wayne T Lamoreaux, Robert K Fairbanks, John J Demakas, Barton S Cooke, Benjamin J Peressini, John T Holbrook, Christopher M Lee

**Affiliations:** 1 Gamma Knife of Spokane, 910 W 5th Ave, Suite 102, Spokane, WA 99204, USA; 2 Cancer Care Northwest, 910 W 5th Ave, Suite 102, Spokane, WA 99204, USA; 3 MacKay & Meyer MDs, 711 S Cowley St, Suite 210, Spokane, WA 99202, USA; 4 Spokane Brain & Spine, 801 W 5th Ave, Suite 210, Spokane, WA 99204, USA; 5 DataWorks Northwest, LLC, 3952 N Magnuson St, Coeur D'Alene, ID 83815, USA

**Keywords:** Brain metastases, Survival, Treatment regimen, Age, Performance status, Primary tumor histology, Tumor number, Tumor volume

## Abstract

**Background:**

Whole brain radiation therapy (WBRT), surgical resection, stereotactic radiosurgery (SRS), and combinations of the three modalities are used in the management of patients with metastatic brain tumors. We present the previously unreported survival outcomes of 275 patients treated for newly diagnosed brain metastases at Cancer Care Northwest and Gamma Knife of Spokane between 1998 and 2008.

**Methods:**

The effects treatment regimen, age, Eastern Cooperative Oncology Group-Performance Status (ECOG-PS), primary tumor histology, number of brain metastases, and total volume of brain metastases have on patient overall survival were analyzed. Statistical analysis was performed using Kaplan-Meier survival curves, Andersen 95% confidence intervals, approximate confidence intervals for log hazard-ratios, and multivariate Cox proportional hazard models.

**Results:**

The median clinical follow up time was 7.2 months. On multivariate analysis, survival statistically favored patients treated with SRS alone when compared to patients treated with WBRT alone (p < 0.001), patients treated with resection with SRS when compared to patients treated with SRS alone (p = 0.020), patients in ECOG-PS class 0 when compared to patients in ECOG-PS classes 2 (p = 0.04), 3 (p < 0.001), and 4 (p < 0.001), patients in the non-small-cell lung cancer group when compared to patients in the combined melanoma and renal-cell carcinoma group (p < 0.001), and patients with breast cancer when compared to patients with non-small-cell lung cancer (p < 0.001).

**Conclusions:**

In our analysis, patients benefited from a combined modality treatment approach and physicians must consider patient age, performance status, and primary tumor histology when recommending specific treatments regimens.

## Background

Brain metastases are defined as cancerous lesions in the brain that originate and spread from an extracranial primary cancer. Brain metastases occur in 20 to 40% of patients with systemic cancer and the incidence is growing due to advances in imaging technologies and the treatment of extracranial disease [1]. The site of metastasis often depends on the nearest location of vascular clusters. As a consequence, the most common primary cancers that have the ability to metastasize to the brain are cancers that develop from the lung or breast [2]. However, metastasis to the brain originating from melanoma, colorectal cancer, renal-cell carcinoma, and carcinoma of multiple other origins may also lead to the development of one or more metastatic brain tumors [3]. Due to a large amount of blood flow, the cerebrum accounts for approximately 80% of all brain metastases, while metastases that arise within the cerebellum and brain stem account for the remaining 20% of metastatic tumors [4].

Patients diagnosed with brain metastases have several potential management options and treatment regimens are dependent on the patient's performance status, age, control of primary cancer, presence of extracranial disease, number of brain metastases, size of brain metastases, and location of brain metastases [1,5]. In general, patients with brain metastases have a poor outlook and survive an average of 1 to 2 months when treated with steroid therapy alone [6]. Whole brain radiation therapy (WBRT) has been historically a standard of care for patients with brain metastases. WBRT takes advantage of differences in radiobiology between tumor cells and nervous tissue by targeting rapidly dividing tumor cells in all areas of the brain, while minimizing damage to the adjacent brain tissue [3]. Due to this favorable radiation cell-kill therapeutic ratio, WBRT extends the survival time of patients who undergo treatment to an average of 4 to 7 months [1]. Surgical resection followed by WBRT has proven to be a superior treatment modality than WBRT alone or surgical resection alone for patients with a high performance status (functionally independent and spend no more than 50% of their day in bed) that possess a single, surgically accessible brain metastasis [7-9]. However, surgical resection followed by WBRT is considered an excessive and potentially destructive treatment modality for patients with multiple brain metastases and has not been investigated in a randomized controlled trial [10].

Stereotactic radiosurgery (SRS) is a highly technical form of radiation therapy that delivers a focused dose of radiation to a single volume, while minimizing damage to nearby, critical structures. The patient's skull is immobilized, allowing a controlled dose of radiation to be delivered to a specified target with sub-millimeter precision. There are currently 4 devices utilized for SRS treatment: Gamma Knife (GK) radiosurgery, linear accelerator (LINAC) based treatment, a cyclotron-based proton beam, and cyberknife technology [3]. Although GK remains the "gold standard" of brain radiosurgery, published reports by Andrews et al. [6] and Sneed et al. [11] concluded that patient prognosis did not differ in terms of the method in which SRS was delivered. The evidence assessing the efficacy of SRS in the treatment of patients with brain metastases is continuously increasing due to the fact that it is capable of targeting any area in the brain with accuracy and can be utilized to irradiate multiple lesions during the same clinical treatment setting. For specific patient subsets that have newly diagnosed brain metastases, WBRT alone, SRS alone, SRS with WBRT, SRS with surgical resection, or a combination of the three treatments can be the optimal management approach.

We present a retrospective survival analysis of the 275 patients treated for newly diagnosed brain metastases at Cancer Care Northwest and Gamma Knife of Spokane between 1998 and 2008; including a comprehensive analysis of the effects treatment regimen, age, Eastern Cooperative Oncology Group-Performance Status (ECOG-PS), primary tumor histology, number of brain metastases, and total volume of brain metastases have on patient survival.

## Methods

We analyzed the patient population baseline characteristics and survival of 275 patients treated for newly diagnosed brain metastases at Cancer Care Northwest and Gamma Knife of Spokane (Deaconess Hospital, Spokane, WA) between 1998 and 2008. After obtaining approval from IRB Spokane (IRB 1554) and the University of Washington Human Subjects Division (Human Subjects Application 36306), the following pre-treatment factors were recorded from the patient's medical records: age at first brain metastasis diagnosis, ECOG-PS at first brain metastasis diagnosis, number of brain metastases, primary tumor histology, and total volume of brain metastases at the time of SRS for patients who received SRS, or at an imaging appointment prior to the patients first treatment session for patients who did not receive SRS. Patients were categorized by age at first brain metastasis diagnosis (<65 years and ≥65 years), number of brain metastases at first diagnosis (1 tumor, 2-4 tumors, >4 tumors), primary tumor histology (non-small-cell lung cancer, small-cell lung cancer, breast cancer, melanoma, renal-cell carcinoma, other/unknown primary), total volume of brain metastases in cm^3 ^(2.0, 2.0-3.9, 4.0-5.9, 6.0-7.9, ≥8.0), and ECOG-PS class (0, 1, 2, 3, 4).

Treatment regimens were prescribed based on the patient's performance status, age, control of primary cancer, presence of extracranial disease, number of brain metastases, size of brain metastases, location of brain metastases, and at the discretion of the treating physician. Of the 275 patients, 117 were treated with WBRT alone, 65 were treated with SRS alone, 48 were treated with WBRT with SRS, 11 were treated with surgical resection with WBRT, 15 were treated with surgical resection with SRS, and 19 were treated with surgical resection + WBRT + SRS. SRS was performed using the Leksell ^60^Co Gamma Knife (model C). The prescribed SRS dose to the 50% isodose line was completed in a single treatment and was based on the patient's tumor volume, tumor location, tumor shape, prior radiation treatment, and standard Radiation Therapy Oncology Group (RTOG) guidelines. The median SRS dose was 18 Gy (13 Gy to 22 Gy). For patients receiving WBRT, the median total dose prescribed was 30 Gy (5 Gy to 54 Gy). Length of follow-up was determined as the time interval between the date of first treatment and the date of the most recent clinical encounter or imaging appointment. Period of survival, in months, was based upon the patient's first treatment session.

Kaplan-Meier survival curves were utilized to compare survival differences between the treatment groups, age groups, ECOG-PS groups, tumor volume groups, primary tumor histology groups, and number of brain metastases groups. Andersen 95% confidence intervals for the median survival times of the groups were constructed. Log-rank tests were employed to determine statistically significant differences between the survival curves of each group. Approximate confidence intervals for the log hazard-ratio were calculated using the estimate of standard error. Finally, the Cox proportional hazard was used in a multivariate analysis of the treatment groups, age groups, ECOG-PS groups, and primary tumor histology groups. All statistical analyses were performed using StatsDirect Version 2.5.7 (StatsDirect Ltd., Altrincham, UK) and SigmaPlot Version 11.0 (SYSTAT Software, Inc. San Jose, CA). Statistical significance was set at a p value < 0.05.

## Results

We identified 275 patients treated at Cancer Care Northwest and Gamma Knife of Spokane for newly diagnosed brain metastases. The median patient age was 60 years (29 years to 86 years) at the time of diagnosis. Non-small-cell lung cancer (NSCLC) was the most common primary tumor histology. Patients possessing a single brain metastasis were the largest tumor number category. Of the 275 total patients, ECOG-PS class was not recorded in 162 patients and total tumor volume was not recorded in 151 patients. Table 1 shows the number of patients according to treatment regimen, age, ECOG-PS class, primary tumor histology, number of brain metastases, and tumor volume of brain metastases. The median patient clinical follow-up time was 7.2 months (0.20 months to 117 months).

**Table 1:** Patient population baseline characteristics

Characteristic	WBRT	SRS	WBRT+ SRS	Surgery+ SRS	Surgery + WBRT	Surgery + WBRT + SRS	Total
	**(n = 117)**	**(n = 65)**	**(n = 48)**	**(n = 15)**	**(n = 11)**	**(n = 19)**	**(n = 275)**

**Age at diagnosis, median (range)**	62 (31-86)	61 (37-84)	57.5 (36-79)	57 (29-72)	60 (42-80)	60 (31-86)	60 (29-86)
<65	61	37	38	13	7	12	168
≥65	56	28	10	2	4	7	107
**ECOG-PS**							
0	1	2	5	1	0	0	9
1	29	19	13	3	1	1	66
2	16	6	4	0	0	3	29
3	4	3	0	0	0	0	7
4	0	0	1	0	0	1	2
Unknown	67	35	25	11	10	14	162
**Primary Tumor Histology**							
NSCLC	37	30	22	6	6	11	112
SCLC	18	5	1	1	2	0	27
Breast	20	8	12	0	0	2	42
Melanoma	7	7	3	4	1	3	25
Renal-cell carcinoma	5	1	1	2	0	0	9
Other	26	9	7	2	0	1	45
Unknown	4	5	2	0	2	2	15
**# Brain Metastases**							
1	34	38	16	10	7	12	117
2-4	26	20	16	3	0	6	71
>4	9	2	7	1	0	0	19
Unknown	48	5	9	1	4	1	68
**Tumor Volume (cm**^**3**^**)**							
<2	1	18	8	1	0	2	30
2-3.9	0	16	8	2	0	3	29
4-5.9	0	6	5	3	0	1	15
6-7.9	0	6	9	0	0	2	17
≥8	0	10	11	3	0	9	33
NA/Unknown	116	9	7	6	11	2	151

ECOG-PS = Eastern Cooperative Oncology Group-Performance Status; NSCLC = non-small-cell lung cancer; SCLC = small-cell lung cancer; SRS = stereotactic radiosurgery; WBRT = whole brain radiation therapy

**Figure 1:**
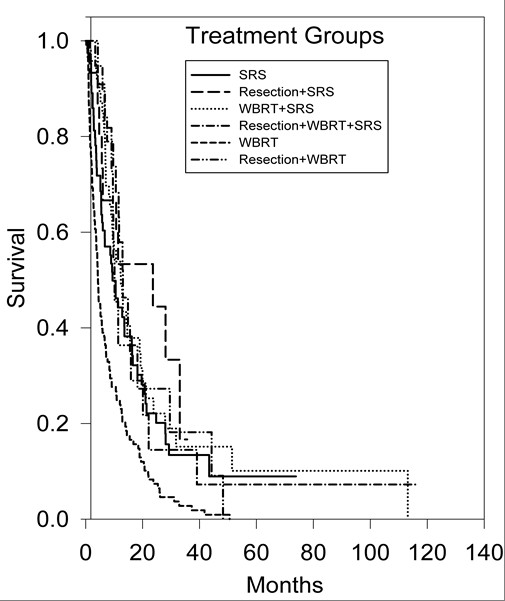
**Kaplan-Meier survival curve illustrating overall survival based on treatment modality**.

**Figure 2:**
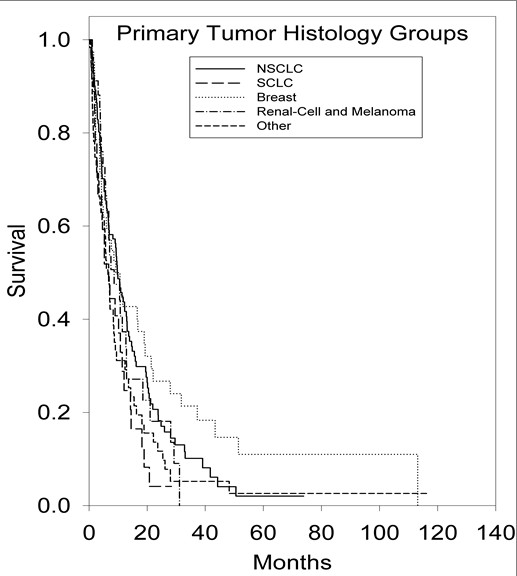
**Kaplan-Meier survival curve illustrating overall survival based on primary tumor histology**.

**Figure 3:**
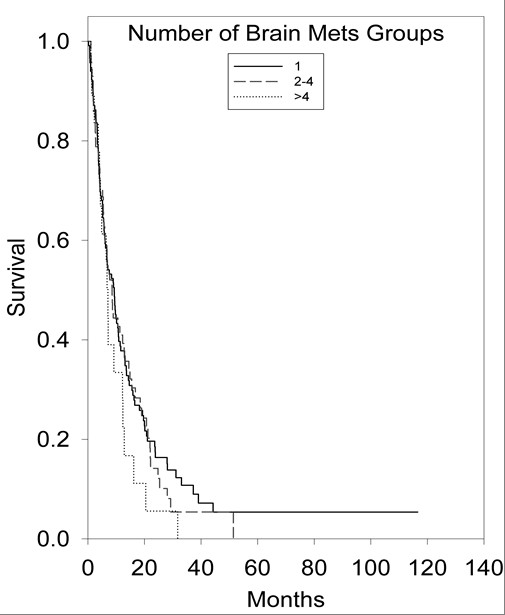
**Kaplan-Meier survival curve illustrating overall survival based on number of brain metastases**.

**Figure 4:**
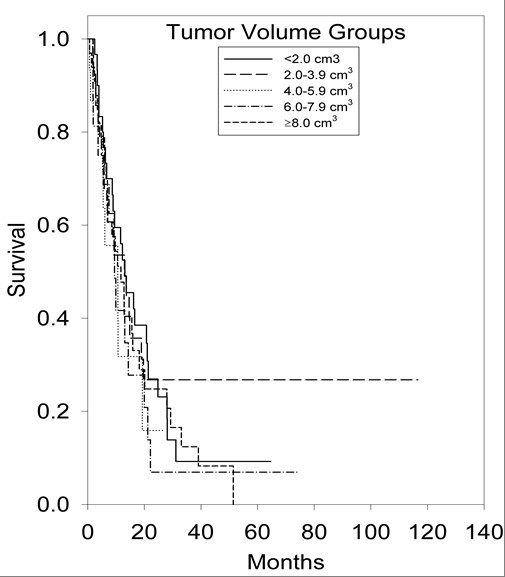
**Kaplan-Meier survival curve illustrating overall survival based on volume of brain metastases**.

An initial statistical analysis was performed using univariate median survival confidence intervals and hazard ratio confidence intervals. Within each category a reference group was selected (treatment regimen = SRS alone, age = less than 65 years, ECOG-PS = 0, primary tumor histology = NSCLC, number of brain metastases = 1, tumor volume = less than 2 cm^3^) and was tested against the other groups' hazard ratios. Univariate hazard ratio analysis of treatment groups indicated that the survival of the SRS alone treatment group was statistically superior (p < 0.001) to the survival of the WBRT alone treatment group (95% CI, 1.37-2.53). Kaplan-Meier survival curves illustrating overall survival based on treatment modality can be found in Figure 1. Univariate hazard ratio analysis of age groups (95% CI, 1.14-1.98) indicated that survival statistically favored patients <65 years of age (p = 0.002). Comparison of univariate hazard ratios in relation to ECOG-PS class indicated that survival statistically favored patients categorized in ECOG-PS class 0 when compared to patients categorized in ECOG-PS class 2 (95% CI, 1.57-6.4) and ECOG-PS class 3 (95% CI, 1.12-15.06), with p values of 0.006 and 0.005, respectively. Comparison of univariate hazard ratios in relation to primary tumor histology indicated that survival statistically favored patients with NSCLC when compared to patients with small-cell lung cancer (SCLC) (95% CI, 0.94-2.61) and patients in the other primary tumor histology group (95% CI, 1.14-2.65), with p values of 0.04 and 0.002, respectively. Kaplan-Meier survival curves illustrating overall survival based on primary tumor histology can be found in Figure 2. The analysis of the number of brain metastases groups and tumor volume groups did not yield any statistically significant results. Kaplan-Meier survival curves showing overall survival based on the number of brain metastases and volume of brain metastases are shown in Figures 3 and 4.

The overall patient median survival time was determined to be 7.9 months. The median survival time for patients treated with WBRT alone was 4.3 months (95% CI, 3.30-5.38), 9.4 months (95% CI, 6.41-12.45) for patients treated with SRS alone, 10 months (95% CI, 8.17-12.15) for patients treated with resection with WBRT, 12 months (95% CI, 8.74-15.98) for patients treated with WBRT with SRS, 13 months (95% CI, 9.70-16.54) for patients treated with resection + WBRT + SRS, and 24 months (95% CI, 1.73-45.55) for patients treated with resection with SRS. Patients <65 years of age survived a median time of 11 months (95% CI, 8.42-12.88), while patients ≥65 years of age survived a median time of 5.7 months (95% CI, 4.29-7.09). The median survival time for patients in ECOG-PS class 0 was 22 months (95% CI, 4.43-39.69), 9.5 months (95% CI, 3.84-15.16) for patients in ECOG-PS class 1, 6.0 months (95% CI, 2.64-9.26) for patients in ECOG-PS class 2, and 1.5 months (95% CI, 0.94-1.96) for patients in ECOG-PS class 3. In regard to primary tumor histology, the median survival time for patients with NSCLC was determined to be 9.78 months (95% CI, 7.90-11.56), 9.2 months (95% CI, 4.04-14.30) for patients with breast cancer, 8.6 months (95% CI, 3.67-13.55) for the combined melanoma and renal-cell carcinoma group, 6.7 months (95% CI, 3.47-10.01) for patients with SCLC, and 5.7 months (95% CI, 2.66-8.72) for patients classified in the other primary tumor histology group.

Further statistical analysis was conducted using multivariate Cox regression analysis with hazard ratio estimates and confidence intervals (Table 2). The multivariate analyses utilized patients treated with SRS alone, patients <65 years of age, patients in ECOG-PS class 0, and patients with NSCLC as the reference groups. Multivariate hazard ratio analysis of treatment groups indicated that the survival of patients in the SRS alone treatment group was statistically superior (p < 0.001) to the survival of the patients in the WBRT alone treatment group (95% CI, 1.37-2.73) and that the survival of the resection with SRS treatment group was statistically superior (p = 0.020) to the survival of the SRS alone treatment group (95% CI, 0.49-0.94). Comparison of multivariate hazard ratios in relation to ECOG-PS class indicated that survival statistically favored patients categorized in ECOG-PS class 0 when compared to patients categorized in ECOG-PS class 2 (95% CI, 1.02-2.72), ECOG-PS class 3 (95% CI, 4.28-4.91), and ECOG-PS class 4 (95% CI, 5.98-21.2), with p values of 0.04, <0.001, <0.001, respectively. Multivariate hazard ratio analysis of primary tumor histology groups indicated that the survival of patients in the breast cancer group was statistically superior (p < 0.001) to the survival of patients in the NSCLC group (95% CI, 0.78-0.96) and that the survival of patients in the NSCLC group was statistically superior (p < 0.001) to the survival of patients in the combined melanoma and renal-cell carcinoma group (95% CI, 1.06-1.3). Multivariate hazard ratio analysis of age groups did not yield any statistically significant results.

**Table 2:** Multivariate hazard ratios, confidence intervals, and p values

	Hazard Ratio		
	
	Estimate	95% CI	p value**
**Treatment Groups**			
SRS*	reference		
Surgery + SRS	0.68	0.49-0.94	0.020
WBRT + SRS	0.99	0.93-1.05	0.660
Surgery + WBRT + SRS	0.79	0.61-1.02	0.070
WBRT	1.94	1.37-2.73	<0.001
Surgery + WBRT	1.04	0.76-1.43	0.800
**Age at diagnosis**			
<65*	reference		
≥65	1.21	0.91-1.62	0.190
**ECOG-PS**			
0*	reference		
1	1.07	0.58-1.95	0.830
2	1.67	1.02-2.72	0.040
3	4.58	4.28-4.91	<0.001
4	11.26	5.98-21.2	<0.001
**Primary Tumor Histology**			
NSCLC*	reference		
SCLC	1.11	0.97-1.26	0.130
Breast	0.87	0.78-0.96	<0.001
Melanoma and Renal-cell	1.17	1.06-1.3	<0.001
Other	1.41	0.95-2.1	0.080

ECOG-PS = Eastern Cooperative Oncology Group-Performance Status; NSCLC = non-small-cell lung cancer; SCLC = small-cell lung cancer; SRS = stereotactic radiosurgery; WBRT = whole brain radiation therapy

* Reference group against which other groups' survival experience are compared

** p value for test if groups' survival experience is same as reference group

## Discussion

Patients with metastatic brain disease have a poor prognosis and curative treatment is not achievable in most clinical situations, with 50% of patients dying from their neurological cancer rather than their extracranial cancer [12]. Due to this unfortunate outlook, maximizing patient's period of survival and comfort level is of great importance. Although several Phase III studies have been published assessing the efficacy of different treatment modalities, many questions still remain unanswered and further randomized evidence is needed not only to prove superior treatments in comparison studies, but to identify optimal courses of treatment in unique patient subsets [6-9,13-17]. Our comprehensive analysis evaluates the clinical effects treatment regimen, age, performance status, primary tumor histology, number of brain metastases, and total volume of brain metastases have on patient survival.

Perhaps the most questionable matter in the management of patients with brain metastases is whether the addition of WBRT to SRS will provide patients with a superior prognosis when compared to patients treated with SRS alone [3]. Our study did not find statistically significant survival differences between the SRS alone treatment group and the SRS with WBRT treatment group in both univariate and multivariate analysis. In the randomized controlled trial published by Aoyama et al. [13], the authors evaluated the clinical outcomes of patients treated with SRS with or without WBRT and also witnessed no significant (p = 0.4) differences in survival between the two treatment arms. However, the patients treated with WBRT with SRS had a substantially better 12-month brain tumor recurrence rate (p < 0.001) and underwent salvage therapy (p < 0.001) less often than the patients treated with SRS alone, but these increases in tumor control did not affect patient survival. Several retrospective cohort studies published in the last ten years have also reported that the addition of WBRT to SRS does not result in superior levels of patient survival [11,18-21].

On multivariate analysis, we found that the survival of the SRS alone treatment arm did not statistically differ when compared to the survival of the resection with WBRT treatment arm. These data correlate with the Phase III randomized trial conducted by Muacevic et al. [17]. A total of 64 patients with a single, surgically accessible brain metastasis ≤30 mm in diameter, a Karnofsky Performance Score (KPS) ≥70, and a controlled primary cancer were randomized into a GK radiosurgery alone group (31 patients) and a surgery with WBRT group (33 patients). The authors reported non-significant differences in survival between the two treatment groups. Rades et al. [22] retrospectively compared SRS alone and surgery with WBRT in 260 patients classified in RPA class 1 or 2 [5] that were diagnosed with 1 to 2 brain metastases and also reported that the two groups did not differ in survival. Our multivariate analysis also found superior levels of survival in patients treated with resection with SRS when compared to patients treated with SRS alone. The body of world literature lacks sufficient studies comparing patients treated with SRS alone against patients treated with resection with SRS. However, survival differences between patients treated with SRS alone and patients treated with resection with SRS was recently reported in another study by Rades et al. [23]. The authors analyzed the clinical outcomes of 164 patients of advanced age (≥65 years). Specifically, 34 patients were treated with WBRT alone, 43 patients were treated with SRS alone, 41 patients were treated with resection + SRS, and 46 patients were treated with resection + WBRT+ SRS boost. In contrast to our results, which favored the resection with SRS treatment group, the authors reported that treatment regimen influenced survival, with the SRS alone treatment group surviving a greater time than the resection + SRS treatment group. The results reported by Rades et al. [23] can be explained when considering the risks of surgery in elderly patients. This data permits the treatment of select patients who are <65 years of age and are functionally independent with resection in combination with SRS.

In subset analysis, patients treated with WBRT alone at our institution exhibited the shortest period of survival, with each of the other five treatment arms surviving a substantially greater time than the WBRT alone treatment arm. Although it is likely that the treatment arms consisted of very different patient subsets with respect to ECOG-PS class, tumor number, tumor volume, and extent of systemic disease, both univariate and multivariate analysis found statistically significant differences between the hazard ratio of patients treated with WBRT and the hazard ratio of patients treated with SRS alone. No randomized controlled trials have been conducted assessing patients treated with SRS alone compared with patients treated with WBRT alone. However, in a recent literature review, Linskey et al. [12] found level 3 evidence indicating that patients with 1 to 3 brain metastases that are treated with SRS alone have superior levels of survival when compared to patients treated with WBRT alone.

As expected, we found that age and performance status are both significant predictors in determining patient prognosis, as survival statistically favored patients <65 years old in univariate analysis and patients in a lower ECOG-PS class in both univariate and multivariate analysis. Several comparison studies have reported a survival dependency on patient age and performance status. Sanghavi et al. [24] retrospectively analyzed the outcomes and potential prognostic factors of a total of 502 patients treated with SRS with WBRT and 1200 patients treated with WBRT alone and found that survival was more pronounced in patients with a higher KPS (p = 0.0001), a controlled primary cancer (p = 0.0023), the absence of extracranial cancer (p = 0.0001), and a lower RPA class (p = 0.000007). Kocher et al. [25] compared the efficacy of SRS alone against WBRT alone in 255 patients with 1 to 3 brain metastases and reported statistically significant increases in median survival in patients categorized in RPA class 1 (p < 0.0001) and RPA class 2 (p < 0.04). Frazier et al. [26] retrospectively analyzed 237 patients treated with SRS ± WBRT and also found that survival statistically favored patients that were <65 years of age (p = 0.008) with KPS values >70 (p = 0.034).

The number and volume of brain metastases patients possess at the time of diagnosis are crucial factors in prescribing the most advantageous course of treatment in select patient groups. When evaluating our six treatment arms in univariate analysis; however, the number and size of brain metastases did not influence patient survival. Tumor resection in combination with WBRT and/or SRS in treating patients with a single brain metastasis is recommended for those who present with severe neurologic deficits, a ventricular obstruction, or a tumor of a large intracranial volume (which often produces mass effect) [1]. When the patient has controlled neurological symptoms, a tumor/s of a small intracranial volume, a single brain metastasis, a surgically inoperable brain metastasis, or multiple brain metastases, SRS alone or in combination with WBRT is often the recommended course of treatment [1]. Questions remain regarding the survival dependency on the number and size of brain metastases patient groups possess. Studies have shown increased survival levels in patients with a single brain metastasis that were treated with radiosurgery [6,26]. However, other publications have reported that tumor volume has a greater impact on patient survival than number of brain metastases and primary tumor histology, with patients possessing small tumor volumes surviving a greater period of time [27-30]. Further study and research is needed on how the number and total volume of brain metastases affect patient survival.

The histologic subtype of the primary tumor may be an essential predictor in assessing the survival advantage of specific patient subsets. NSCLC is known to produce the greatest amount of metastatic brain lesions [31,32]. In univariate analysis, survival statistically favored patients with NSCLC when compared to patients with SCLC and patients classified in the other primary histology group. In multivariate analysis; however, survival statistically favored patients in the breast cancer group when compared to patients in the NSCLC group. Increases in the survival of breast cancer patients when compared to NSCLC patients was also recently reported in the survival analysis of 237 patients treated with radiosurgery by Frazier et al. [26]. These results are likely due to advances in the surgical and chemotherapeutic care of breast cancer patients [33]. It was also observed in multivariate analysis that survival statistically favored patients with NSCLC when compared to the combined melanoma and renal-cell carcinoma group. Traditionally, melanoma and renal-cell carcinoma have been classified as "radioresistant" tumor histologies because of their negative response to standard radiation treatment. However, several studies have reported positive outcomes when treating patients with melanoma and renal-cell carcinoma primaries with radiosurgery [34-40]. In a phase II trial conducted by Manon et al. [41], 31 patients diagnosed with melanoma, renal-cell carcinoma, and sarcoma primary cancers with 1 to 3 brain metastases were treated with SRS alone. The 3 and 6 month intracranial failure rate for the evaluated patients was found to be 25.8 and 48.3%, respectively. The authors concluded that delaying WBRT for patients with melanoma, renal-cell carcinoma, and sarcoma primary cancers may be appropriate for specific subgroups of patients, but must be approached with caution.

## Conclusions

We report retrospectively on the effects treatment regimen, age, performance status, primary tumor histology, number of brain metastases, and volume of brain metastases have on the survival of patients diagnosed with brain metastases. Multivariate analysis of treatment regimens showed that survival statistically favored patients treated with SRS alone and patients treated with resection with SRS when compared to patients treated with WBRT alone and patients treated with SRS alone, respectively. Comparison of multivariate hazard ratios in relation to ECOG-PS class indicated that survival statistically favored patients categorized in ECOG-PS class 0 when compared to patients categorized in ECOG-PS classes of 2, 3, and 4. Multivariate analysis of primary tumor histology groups indicated that the survival of patients in the breast cancer group was statistically superior to the survival of patients in the NSCLC group and that the survival of patients in the NSCLC group was statistically superior to the survival of patients in the combined melanoma and renal-cell carcinoma group. In our analysis, patients benefited from a combined modality treatment approach and physicians must consider patient age, performance status, and primary tumor histology when recommending specific treatment regimens.

## Competing interests

The authors declare that they have no competing interests.

## Authors' contributions

ALE and CML reviewed relevant literature and drafted the manuscript. BJP conducted all statistical analyses. ARM, WTL, RKF, JJD, BSC, and JTH provided clinical expertise and participated in drafting the manuscript. All authors read and approved the final manuscript.
